# Molecular and Morphological Analyses Reveal Phylogenetic Relationships of Stingrays Focusing on the Family Dasyatidae (Myliobatiformes)

**DOI:** 10.1371/journal.pone.0120518

**Published:** 2015-04-13

**Authors:** Kean Chong Lim, Phaik-Eem Lim, Ving Ching Chong, Kar-Hoe Loh

**Affiliations:** 1 Institute of Biological Sciences, University of Malaya, Kuala Lumpur, Malaysia; 2 Institute of Ocean and Earth Sciences, University of Malaya, Kuala Lumpur, Malaysia; University of Minnesota, UNITED STATES

## Abstract

Elucidating the phylogenetic relationships of the current but problematic Dasyatidae (Order Myliobatiformes) was the first priority of the current study. Here, we studied three molecular gene markers of 43 species (COI gene), 33 species (ND2 gene) and 34 species (RAG1 gene) of stingrays to draft out the phylogenetic tree of the order. Nine character states were identified and used to confirm the molecularly constructed phylogenetic trees. Eight or more clades (at different hierarchical level) were identified for COI, ND2 and RAG1 genes in the Myliobatiformes including four clades containing members of the present Dasyatidae, thus rendering the latter non-monophyletic. The uncorrected p-distance between these four ‘Dasytidae’ clades when compared to the distance between formally known families confirmed that these four clades should be elevated to four separate families. We suggest a revision of the present classification, retaining the Dasyatidae (*Dasyatis* and *Taeniurops* species) but adding three new families namely, Neotrygonidae (*Neotrygon* and *Taeniura* species), Himanturidae (*Himantura* species) and Pastinachidae (*Pastinachus* species). Our result indicated the need to further review the classification of *Dasyatis microps*. By resolving the non-monophyletic problem, the suite of nine character states enables the natural classification of the Myliobatiformes into at least thirteen families based on morphology.

## Introduction

The family Dasyatidae in the Order Myliobatiformes is one of the biggest families of batoid fishes. According to Carpenter & Niem [1], the body of members of the dasyatids is characterized by a large, oval, circular or rhomboidal disc usually covered with denticles, thorns and tubercles on the dorsal surface and sometimes on the tail. Given the large number of species described in the Dasyatidae, the classification and status of the described species are still in flux owing to taxonomic uncertainties especially at the family level. The few comprehensive studies on the classification within the Dasyatidae are based either on morphology, including their external morphological structures, squamation, tooth root vascularization and structure, lateral line canal, skeletal structure and cephalic and branchial musculature [2], or molecular markers including mtGenome, RAG1 and SCFD2 [3, 4]. Nevertheless, these approaches fail to classify the Dasyatidae such as the species of *Himantura* and *Pastinachus* into defined clusters since some still remain as *incertae sedis* or uncertain placements. The binary differentiation based on the absence and presence of placoid scales (or more often terms such as thorns and denticles) as adopted by McEachran & Aschliman [2] is thought to be too general because there are instances of variable patterns of thorns and denticles among the dasyatids. On the other hand, some distinct characters such as the ventral tail fold and body and tail pigmentations, not included in McEachran & Aschliman [2], may be used to resolve the taxonomic uncertainties between *Himantura* and *Pastinachus* [5].

DNA barcoding using the Cytochrome Oxidase I (COI) gene has been widely used to identify fishes and other organisms at the species level [6–10]. In addition, COI gene also enables researchers to separate phylogeographically distinct populations within species [6]. Another mitochondrial gene, NADH dehydrogenase 2 (ND2), has also been used to construct a phylogenetic tree of sharks and rays with high taxonomic certainty [11]. Thus, the present search of the nucleotides sequences available in NCBI GenBank has indicated that both COI and ND2 genes constitute the highest number of sequences among batoids. The search also found two available nuclear DNA genes for batoids, Sec1 Family Domain-Containing Protein 2 (SCFD2) and Recombination Activating Gene 1 (RAG1), but only the latter is available for a reasonable number of batoid species to be of use in a phylogenetic analysis. Given the availability of these sequences, a molecular approach based on COI, ND2 and RAG1 genes may be useful to evaluate the batoids at the family level, and resolve the uncertainties that clouded the current taxonomy of the Dasyatidae.

The aims of the present study were therefore to elucidate the phylogenetic relationships of the Dasyatidae stingrays using COI, ND2 and RAG1 genes, and to re-examine the morphological characters used by Last *et al*. [5] for species differentiation.

## Methods

### Ethics Statement

No specific permits were required for the described field studies. The stingrays were collected from fish markets and none of the collected specimens were in ‘Endangered’ category of IUCN Red List. The specimens collected at fish market were dead upon purchasing.

### Sampling

Stingray samples belonging to the Order Myliobatiformes were collected from fish markets in Sandakan, Tawau, Semporna and Kuala Selangor, Malaysia. Twenty-two species of stingrays which included 16 species of dasyatids were identified based on Last *et al*. [5]. Three to five fin clips or tissue samples from each species were taken and immediately put into 99.8% ethanol for genetical/DNA analysis. All voucher specimens of small individuals were kept in B201 Environmental Laboratory, Institute of Postgraduate Studies, University of Malaya, as reference collections.

### Laboratory procedures

COI [8], ND2 [11] and RAG1 [4] genes were used in the phylogenetic analysis. However, only the determination of COI and ND2 followed the laboratory procedures described below, while the RAG1 gene sequences were extracted entirely from NCBI Genbank.

DNA was extracted using G-spin Total DNA Extraction Mini Kit (iNtRON Biotechnology, Inc, Korea). Both COI and ND2 genes were amplified by polymerase chain reaction (PCR) using the universal primer FishF2 (5’TCG ACT AAT CAT AAA GAT ATC GGC AC3’) and FishR2 (5’ACT TCA GGG TGA CCG AAG AAT CAG AA 3’) for COI gene [7] and ILEM (5’ AAG GAG CAG TTT GAT AGA GT 3’) and ASNM (5’ AAC GCT TAG CTG TTA ATT AA 3’) for ND2 gene [11]. The PCR cocktail containing 2 μL of 10x PCR buffer, 2μL of dNTPs mixture (2.5mM each), 1 μL of 10pmol primer (both primers), 1.0 unit of *Taq* DNA polymerase, 50pg to 1.0μg DNA templates, and UHQ water was added to a final volume of 20μL. The PCR cycles for COI gene comprised of 4 min initial denaturation at 94°C, followed by 30 cycles of 1 min at 94°C, 0.45 min at 50°C, 1 min at 72°C and with final extension of 10 min at 72°C. For ND2, the modification on the PCR cycles included 30 cycles of 30 sec at 94°C, 30 sec at 50°C, 1min at 72°C and with final extension of 10 min at 72°C. The PCR products were examined using 1% agarose in TAE buffer. All samples that showed good PCR amplifications were sent for sequencing after purification using LaboPass Gel & PCR Purification Kit (Cosmo Genetech, South Korea). The obtained sequencing results were preliminary checked for confirmation of species using Blastn tool of NCBI. Sequences used for the phylogenetic analysis were submitted to GenBank database with accession numbers as in S1 Table.

### Sequences analysis

DNA sequences were aligned and trimmed using ClustalX [12] and BioEdit software [13] respectively. The aligned sequences were subjected to the best model search for Bayesian Inference (BI) and Maximum Likelihood (ML) analyses using Kakusan v. 3 [14]. The generated files were subsequently used for phylogenetic trees construction using Mr Bayes for BI [15] and Treefinder for ML [16]. The selected model for ML was J1 + Gamma (COI gene), J2 + Gamma (ND2 gene) and J1 + Gamma (RAG1 gene) based on Akaike Information Criterion (AIC). ML analyses were performed with 1000 bootstrap replicates. The selected model for BI was HKY85 + Gamma (COI, ND2 and RAG1 genes) based on Bayesian Information Criterion (BIC). Bayesian analyses were initiated with a random starting tree and two parallel runs, each of which consisted of running four chains of Markov chain Monte Carlo (MCMC) iterations for 2000000 generations. The trees in each chain were sampled every 200th generation. Likelihood values for all post-analysis trees and parameters were evaluated for convergence and burn-in using the “sump” command in MrBayes and 200 trees were discarded as burn-in (where the likelihood values were stabilized prior before the burn in), and the remaining trees after burn-in were used to calculate posterior probabilities using the “sumt” command.

The resulting ML and BI phylogenetic trees were processed via Figtree v1.3.1 (http://tree.bio.ed.ac.uk/software/figtree/). In the phylogenetic analyses, the sharks, *Carcharhinus plumbeus* (EU398639 for COI, JQ518632 for ND2 and AY462152 for RAG1) and *Carcharhinus amblyrhynchos* (EF609308 for COI and JQ519095 for ND2) from GenBank were used as outgroups. For all genes, phylograms were constructed if feasible, if not, a cladogram. Some other sequences of same or closely related species of stingrays (in the Order Myliobatiformes) from GenBank were also used in the tree construction for comparison. The accession number of sequences from NCBI Genbank and other information regarding number and percentage of species sampled for this study are given in S2 Table. Uncorrected p-distance was calculated using PAUP* 4.0b10 software [17] to observe the genetic divergence between stingray clusters.

### Character identification

To reclassify the dasyatid species, especially *Himantura* and *Pastinachus*, the morphological characters used in previous taxonomic keys on sharks and rays [1, 2, 5, 18–20] were examined, selected and modified according to the findings from the present study. Selection of representative species from all families within Order Myliobatiformes was based on the species descriptions available in the literatures (S2 Table) [1, 2, 5, 18–24]. The selected morphological characters used in the present study included body or disc shape, body thorns and denticles, head position and elevation, snout or rostral fin form, gill openings, tail types, tail colour pattern, ventral tail fold and caudal fin. Nine character states were constructed. Each state was assigned numeric codes (0–3) to define a set of distinguishing morphological characters. The character score of each representative species was recorded based on the available descriptions of published works [1, 5, 18–24]. The scored character states were then used to construct a character matrix. Both morphology (based on the character matrix) and molecular information (based on the constructed phylogenetic tree) were then combined to obtain a robust dichotomous key that attempts to improve the current classification key to the families of the Myliobatiformes stingrays [1].

To test the robustness and functionality of the constructed classification key, 17 other species not used in the construction of the character matrix were sampled from the Dasyatidae [5, 18, 20–23, 25, 26]. The required character states were then extracted from these species to form the test character matrix which was then used to test the accuracy of the classification key.

### Morphometric analysis

Morphometric data of 27 characters that were used to describe members of the Dasyatidae (including proposed new families) were compiled from various references [21, 27–31] including new measurements from the present sampling (see S4 Table). The available morphometric data included those from 19 species. The percentage to disc width (DW) of each measurement for each family was calculated to provide the maximum, minimum, mean and standard deviation of the measurement. These values were compared between families to aid in their classification.

Fifteen morphometric characters that were present in the four examined ‘dasyatid’ families were further analysed using forward stepwise discriminant analysis (SDFA) in the software Statistica version 8.0 [32]. The SFDA extracts the minimum number of morphometric characters that will effectively distinguish the families. Default tolerance setting was retained at 0.10, with F to enter = 3 and F to exit = 2.

## Results

### Phylogenetic analysis

**Fig 1:**
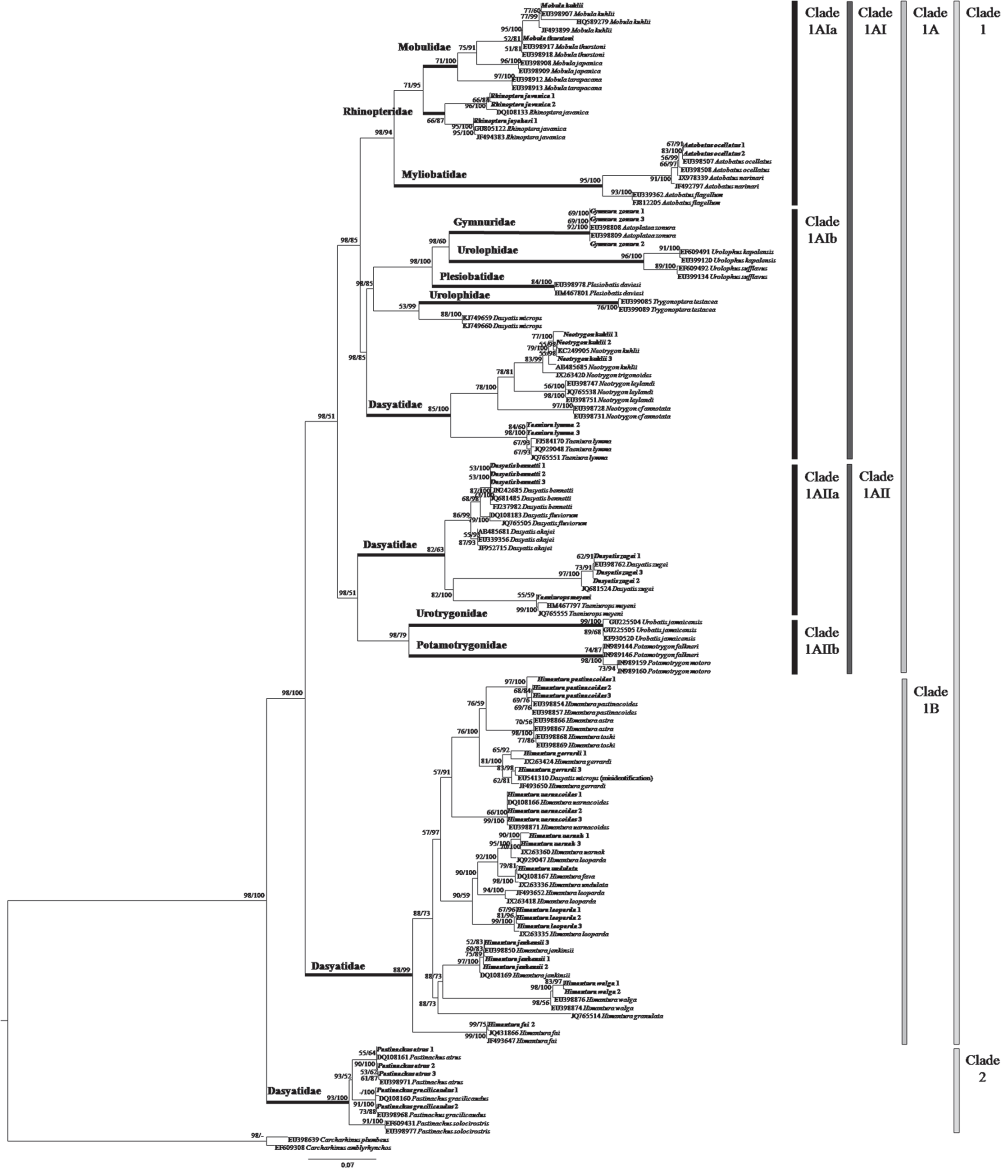
COI gene phylogenetic relationships of stingrays (phylogram). The bootstrap values (ML/Bayesian Inference) are shown at branches.

**Fig 2:**
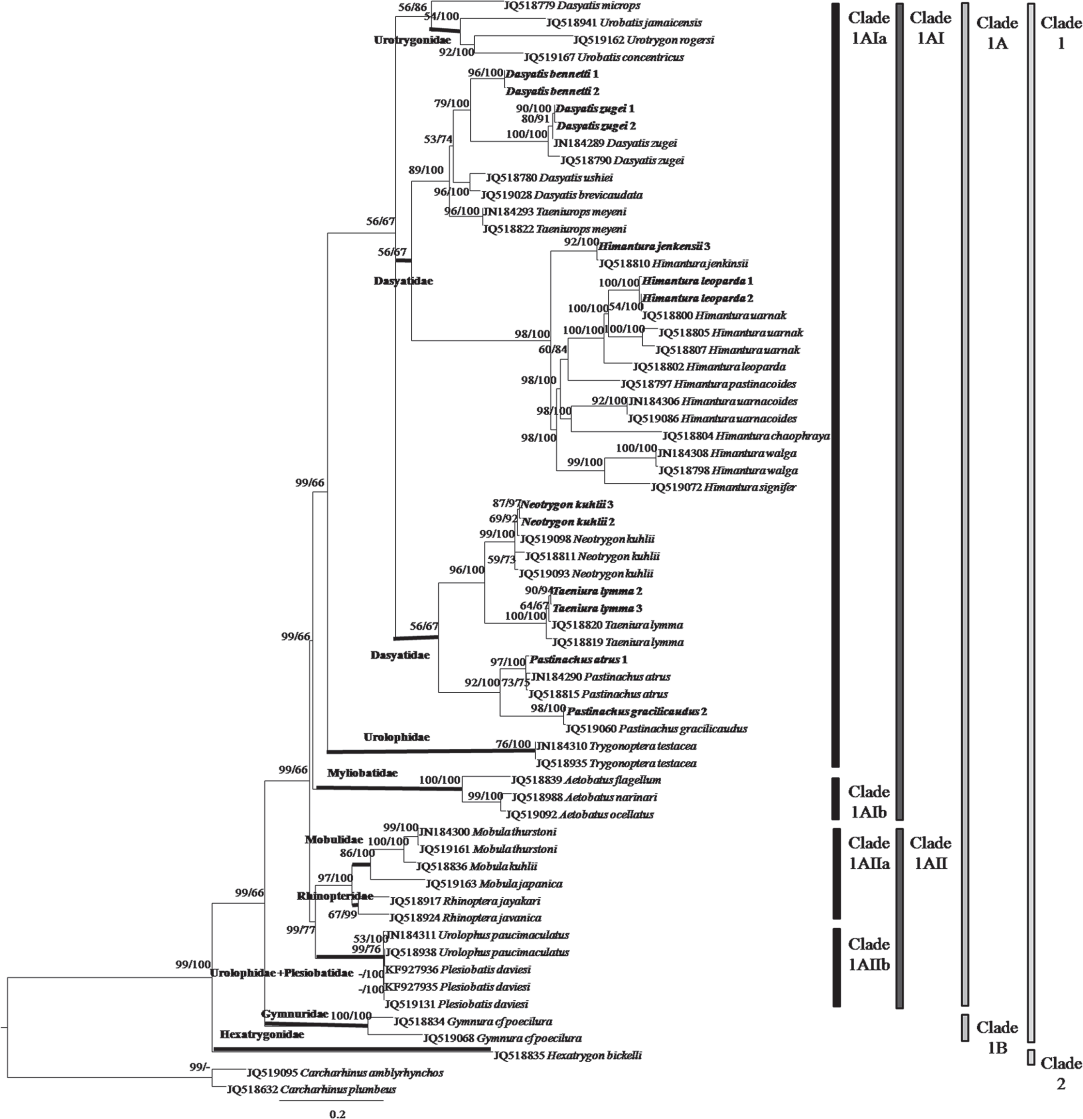
ND2 gene phylogenetic relationships of stingrays (phylogram). The bootstrap values (ML/Bayesian Inference) are shown at branches.

**Fig 3:**
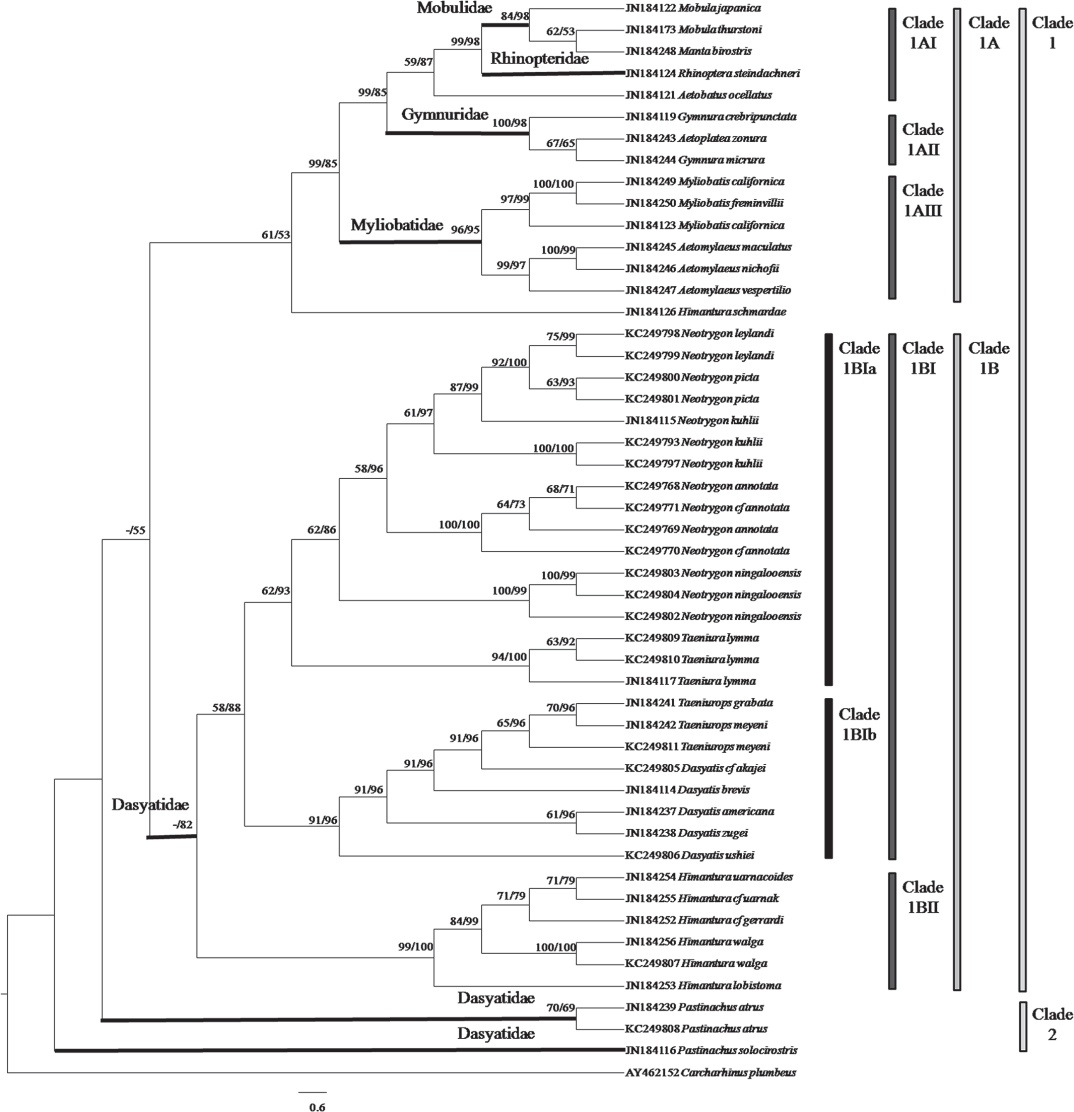
RAG1 gene phylogenetic relationships of stingrays (cladogram). The bootstrap values (ML/Bayesian Inference) are shown at branches.

A total of 47 tissue samples belonging to 5 families and 22 species of stingrays were used for the COI analysis. For ND2 gene, the analysis aimed to clarify the current ‘Dasyatidae’ at the familial level which involved 13 tissue samples from four possible clusters within the family. Another 42 species (COI gene) and 32 species (ND2 gene) of similar or closely related species within the Order Myliobatiformes were included in the phylogenetic analysis (Figs 1 & 2). As for RAG1 gene, the phylogenetic analysis was based on NCBI Genbank sequences of 34 species of stingrays within the Order Myliobatiformes (Fig 3). As shown in all phylogenetic trees, families of the stingrays were not monophyletic (Figs 1 and 2 & 3).

The ML and BI combined tree for COI gene (Fig 1) showed that the Myliobatiformes members could be divided into two major clades: Clade 1 (Myliobatidae, Gymnuridae, Mobulidae, Rhinopteridae, Urolophidae, Plesiobatidae, Urotrygonidae, Potamotrygonidae, Dasyatidae (*Neotrygon*, *Taeniura*, *Dasyatis*, *Taeniurops* and *Himantura* species)) and Clade 2 (*Pastinachus* species) with high and full support bootstrap value (ML/BI = 98/100%). Clade 1 was divided into two subclades: Clade 1A (Myliobatidae, Gymnuridae, Mobulidae, Rhinopteridae, Urolophidae, Plesiobatidae, Urotrygonidae, Potamotrygonidae, Dasyatidae (*Neotrygon*, *Taeniura*, *Dasyatis* and *Taeniurops* species)) and Clade 1B (*Himantura* species) with high and full support bootstrap value (98/100%). Clade 1A was further subdivided into Clade 1AI (Myliobatidae, Gymnuridae, Mobulidae, Rhinopteridae, Urolophidae, Plesiobatidae, and Dasyatidae (*Neotrygon* and *Taeniura* species)) and Clade 1AII (Urotrygonidae, Potamotrygonidae, *Dasyatis* and *Taeniurops* species) with high and weak support bootstrap value (98/51%). Clade 1AI was subdivided into Clade 1AIa (Myliobatidae, Mobulidae, and Rhinopteridae) and Clade 1AIb (Gymnuridae, Urolophidae, Plesiobatidae, *Neotrygon* and *Taeniura* species) with high and moderate support bootstrap value (98/85%). Clade 1AII was further subdivided into Clade 1AIIa (*Dasyatis* and *Taeniurops* species) and Clade 1AIIb (Urotrygonidae and Potamotrygonidae) with high and weak support bootstrap value (98/51%).

The ML and BI combined tree for ND2 gene (Fig 2) showed that the Myliobatiformes members could be divided into two clades: Clade 1 (Mobulidae, Myliobatidae, Rhinopteridae, Plesiobatidae, Urolophidae, Urotrygonidae, Gymnuridae, and Dasyatidae) and Clade 2 (Hexatrygonidae), had high and full support bootstrap values (ML/BI = 99/100%). Clade 1 was subdivided into Clade 1A (Mobulidae, Myliobatidae, Rhinopteridae, Plesiobatidae, Urolophidae, Urotrygonidae, and Dasyatidae) and Clade 1B (Gymnuridae), with high and weak support bootstrap values (99/66%). Clade 1A was further subdivided into Clade 1AI (Myliobatidae, Urotrygonidae, Urolophidae (*Trygonoptera* species) and Dasyatidae) and Clade 1AII (Mobulidae, Rhinopteridae, Plesiobatidae and Urolophidae (*Urolophus* species) with high and weak support bootstrap values (99/66%). Clade 1AI was then subdivided into Clade 1AIa (Urotrygonidae, Urolophidae (*Trygonoptera* species), and Dasyatidae) and Clade 1AIb (Myliobatidae) with high and weak support bootstrap values (99/66%).

For RAG1 gene, a cladogram was used since the distances among the taxa were small resulting in a crowded phylogenetic tree (not shown). The ML and BI combined tree for RAG1 gene (Fig 3) showed that the Myliobatiformes members could be divided into two major clades: Clade 1 (Mobulidae, Rhinopteridae, Gymnuridae, Myliobatidae and Dasyatidae (*Neotrygon*, *Taeniura*, *Dasyatis*, *Taeniurops and Himantura* species)) and Clade 2 (*Pastinachus*). Clade 1 was divided into two subclades: Clade 1A (Mobulidae, Rhinopteridae, Gymnuridae and Myliobatidae) and Clade 1B (*Neotrygon*, *Taeniura*, *Dasyatis*, *Taeniurops* and *Himantura* species) with slightly weak support bootstrap value (-/55%). Clade 1A was further subdivided into Clade 1AI (Mobulidae and Rhinopteridae), Clade 1AII (Gymnuridae) and Clade 1AIII (Myliobatidae) with high support bootstrap value (99/85%). Clade 1B was subdivided into Clade 1BI (*Neotrygon*, *Taeniura*, *Dasyatis* and *Taeniurops* species) and Clade 1BII (*Himantura* species) with high support bootstrap value (-/82%). Lastly, Clade 1BI was divided into two subclades: Clade 1BIa (*Neotrygon* and *Taeniura* species) and Clade 1BIb (*Dasyatis* and *Taeniurops* species) with weak and high support bootstrap value (58/88%).

The phylogenetic trees clearly showed that members of Dasyatidae were not monophyletic forming four clades in COI and RAG1 genes, and two main clades each with two subclades in ND2. The four clades or subclades include: a. *Neotrygon* and *Taeniura* species, b. *Dasyatis* and *Taeniurops* species, c. *Himantura* species, and d. *Pastinachus* species. From the COI phylogenetic tree, the two genera *Neotrygon* and *Taeniura* showed sister relationships and were grouped with three other families of Myliobatiformes including Gymnuridae, Urolophidae and Plesiobatidae.

The range of uncorrected p-distance among families was identified by comparing the distances among formally known families (sensu [1]) in the Order Myliobatiformes (Myliobatidae, Gymnuridae, Mobulidae, Rhinopteridae, Plesiobatidae, Urolophidae, Urotrygonidae, Potamotrygonidae, Dasyatidae and Hexatrygonidae). The p-distances among families ranged from 11.00 to 24.88%, 12.54 to 30.29% and 2.94 to 9.29% for COI, ND2 and RAG1 genes, respectively (Table 1). The genetic distance between *Rhinoptera* and other families was inconclusive in RAG1 gene as there was only one sequence available for *Rhinopotera* species and therefore should be ignored. Using the p-distance range in Table 1, distances between the four clusters within the Dasyatidae (as shown in Figs 1 and 2 & 3) were compared to determine whether each of these clusters should be considered as single family (Table 2). The p-distances of the four clusters ranged from 14.11 to 22.21%, 15.62 to 23.44% and 2.38 to 9.08% for COI, ND2 and RAG1 genes, respectively. These distances were comparable to the range of p-distances computed for the known families of the Myliobatiformes (Table 1), and therefore, the current Dasyatidae should be split into four families. The available sequences of *Dasyatis microps* in COI gene (accession number KJ749659, KJ749660 and EU541310 (misidentified)) and ND2 gene (accession number JQ518779) were found to be distant from other members of *Dasyatis*, with p-distances that ranged from 14.42 to 17.38% and 16.03 to 19.68% for COI and ND2 genes, respectively. Its distance from other members of Myliobatiformes was also found to be high, ranging from 13.08 to 18.25% and 17.85 to 24.00% for COI and ND2 genes, respectively. In addition, the position of *D*. *microps* in both COI and ND2 genes was placed away from other *Dasyatis* but formed the cluster with Urolophidae (*Trygonoptera* species) in COI gene and Urotrygonidae in ND2 gene. Therefore, the current taxonomic classification of *D*. *microps* should be revised.

**Table 1:** Range of uncorrected p-distances for COI, ND2 and RAG1 genes among families in the Order Myliobatiformes.

Family	Family	COI	ND2	RAG1
Myliobatidae	Mobulidae	17.97–23.96	22.16–23.41	2.94–4.55
Myliobatidae	Rhinopteridae	16.87–19.63	21.51–22.86	2.74–4.40
Myliobatidae	Gymnuridae	17.09–18.90	22.83–24.37	3.14–5.32
Myliobatidae	Plesiobatidae	19.94–21.35	21.32–22.18	-
Myliobatidae	Urolophidae	19.05–21.50	22.73–26.32	-
Myliobatidae	Urotrygonidae	17.64–20.03	21.61–24.02	-
Myliobatidae	Potamotrygonidae	19.06–21.05	-	-
Myliobatidae	Dasyatidae	17.33–22.61	21.45–26.40	3.15–9.29
Myliobatidae	Hexatrygonidae	-	25.54–25.73	-
Mobulidae	Rhinopteridae	11.00–17.48	12.54–14.65	0.97–1.66
Mobulidae	Gymnuridae	18.46–22.70	20.71–23.41	3.68–4.49
Mobulidae	Plesiobatidae	16.65–22.70	19.10–19.48	-
Mobulidae	Urolophidae	16.73–24.88	21.76–24.97	-
Mobulidae	Urotrygonidae	18.10–23.01	18.71–22.57	-
Mobulidae	Potamotrygonidae	15.48–22.31	-	-
Mobulidae	Dasyatidae	14.33–23.47	18.22–24.37	4.28–8.42
Mobulidae	Hexatrygonidae	-	25.24–26.40	-
Rhinopteridae	Gymnuridae	17.48–19.01	20.16–22.28	3.47–4.04
Rhinopteridae	Plesiobatidae	16.07–16.99	17.96–18.43	-
Rhinopteridae	Urolophidae	17.20–20.27	21.33–22.09	-
Rhinopteridae	Urotrygonidae	16.87–18.71	17.28–20.76	-
Rhinopteridae	Potamotrygonidae	17.34–18.28	-	-
Rhinopteridae	Dasyatidae	14.23–20.09	16.40–23.43	3.85–7.80
Rhinopteridae	Hexatrygonidae	-	26.14–26.53	-
Gymnuridae	Plesiobatidae	17.41–17.64	20.64–22.57	-
Gymnuridae	Urolophidae	18.29–19.40	23.23–25.55	-
Gymnuridae	Urotrygonidae	18.71–19.58	18.42–24.40	-
Gymnuridae	Potamotrygonidae	18.27–18.91	-	-
Gymnuridae	Dasyatidae	15.69–20.86	19.72–26.40	4.54–9.09
Gymnuridae	Hexatrygonidae	-	25.05–26.40	-
Plesiobatidae	Urolophidae	17.33–18.32	21.45–22.83	-
Plesiobatidae	Urotrygonidae	19.33–20.23	19.58–21.99	-
Plesiobatidae	Potamotrygonidae	19.21–19.64	-	-
Plesiobatidae	Dasyatidae	16.87–21.86	17.83–24.43	-
Plesiobatidae	Hexatrygonidae	-	24.64	-
Urolophidae	Urotrygonidae	19.33–22.76	22.25–25.64	-
Urolophidae	Potamotrygonidae	17.67–21.33	-	-
Urolophidae	Dasyatidae	16.69–23.48	20.99–27.86	-
Urolophidae	Hexatrygonidae	-	26.95–28.68	-
Urotrygonidae	Potamotrygonidae	17.17–18.75	-	-
Urotrygonidae	Dasyatidae	17.04–21.96	15.66–23.63	-
Urotrygonidae	Hexatrygonidae	-	25.24–27.67	-
Potamotrygonidae	Dasyatidae	14.95–20.59	-	-
Potamotrygonidae	Hexatrygonidae	-	-	-
Dasyatidae	Hexatrygonidae	-	23.56–30.29	-

**Table 2:** Range of uncorrected p-distances among the four clusters in the Dasyatidae (sensu Carpenter & Niem, 1999) based on COI, ND2 and RAG1 genes.

Cluster	Cluster	COI	ND2	RAG1
Dasyatis	Neotrygon	14.11–19.01	15.65–20.44	2.38–5.40
Dasyatis	Himantura	16.56–22.21	18.46–23.44	5.89–7.36
Dasyatis	Pastinachus	14.42–20.75	15.62–20.46	4.14–6.49
Neotrygon	Himantura	15.18–20.18	19.65–23.22	6.76–9.08
Neotrygon	Pastinachus	15.03–17.28	17.05–18.50	5.30–7.86
Himantura	Pastinachus	15.49–19.27	19.27–22.76	7.35–8.69

### Proposed new families and reclassification of Order Myliobatiformes

The derived family character matrix that uniquely distinguishes the thirteen families in the Myliobatiformes included three newly proposed families (Table 3). The family character matrix was constructed from the character states of 47 sampled species (S3 Table). We have included *Dasyatis microps* as a separate, additional ‘family’ due to its uniqueness. Hence, the nine character states which distinguish the families could be used to construct a classification key to the stingray families as given below.

**Table 3:** Character matrix for thirteen major families (including *Dasyatis microps*) of the Myliobatiformes (present study) based on nine character states.

	Character states								
Family	1	2	3	4	5	6	7	8	9
Mobulidae	0	0	0	2	1	1	0	0	0
Rhinopteridae	0	0	0	1	1	1	0	0	0
Myliobatidae	0	0	0	0	1	1	0	0	0
Gymnuridae	0	0	1	3	1	1	1	0	0
Plesiobatidae	1	2	1	3	1	0	0	0	1
Hexatrygonidae	1	0	1	3	0	0	0	0	1
Urolophidae	1	0	1	3	1	0	0,1	0	1
Urobatidae	1	0,3	1	3	1	0	1	0	1
Potamotrygonidae	1	3	1	3	1	0	1	3	0
Himanturidae*	1	2	1	3	1	0,1	0,1	0	0
Pastinachidae*	1	2	1	3	1	1	0	2	0
Neotrygonidae*	1	1	1	3	1	0	1	1	0
Dasyatidae	1	0,1	1	3	1	0,1	0	1	0
*“Dasyatis microps”*	1	3	1	3	1	1	0	0	0

* Proposed new families

**Character 1**: Body disc shape: 0 = wing like; pectoral fin greatly expanded, 1 = rhombus, quadrangular or oval; pectoral fin not greatly expanded. **Character 2**: Body denticles and thorns: 0 = no distinct denticles and thorns, 1 = no distinct denticles; thorn confined to midline of disc, 2 = granular or flat denticles band very broad; some may have thorns that either confine to center of body or midline, thorns can be blunt or sharp, 3 = with small spiny or star like denticles; no thorns along central disc or tail. **Character 3**: Head position and elevation: 0 = head extended anterior to pectoral fin; head elevated, 1 = head not extended anterior to pectoral fin; head not elevated. **Character 4**: Rostrum or cephalic fin: 0 = rostral fin single and convex, 1 = rostral fin bilobate and broadly notched medially 2 = Snout forming bilobate cephalic fin, laterally based on head, 3 = not as stated. **Character 5**: Gill opening: 0 = six gill opening, 1 = 5 gill opening. **Character 6**: Tail types: 0 = tail short and stout, not whip like, 1 = tail long, whip like. **Character 7**: Tail pattern: 0 = plain, 1 = banded or striped. **Character 8**: Ventral skin fold: 0 = no ventral skin fold, 1 = low ventral skin fold, with or without indistinct dorsal skin fold, 2 = large ventral skin fold, 3 = distinct dorsal and ventral skin fold. **Character 9**: Caudal fin: 0 = no caudal fin, 1 = with well developed caudal fin.

### Morphometric analysis and descriptions of Dasyatidae with proposed new families

**Fig 4:**
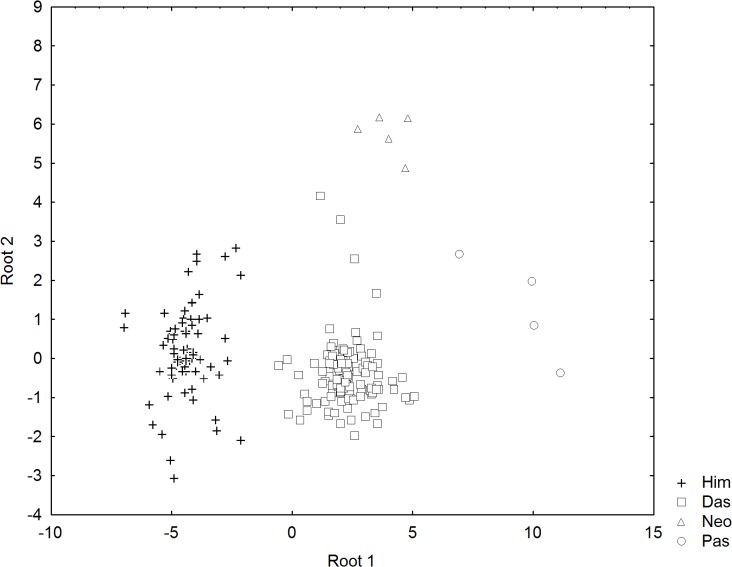
Plots of canonical scores derived from forward stepwise discriminant analysis of morphometric measurements of four stingray families. Squares = Dasyatidae; triangles = Neotrygonidae; crosses = Himanturidae; circles = Pastinachidae.

The SDFA of the morphometric measurements indicated that the four families of dasyatids could be distinguished based on the first two canonical roots which explained 96.0% of the total variation. Nine character measurements were identified as the most useful in the SDFA model, including relative total length (TL), disc length (DL), tail width (TW), tail height (TH), eye diameter (ED), spiracle length (SPL), interspiracular length (ISL), distance between fifth gill slits (I5) and ventral tail fold length (VFL). The biplot of canonical scores of these characters on the first two roots show four separable clusters each belonging to a family (Fig 4). On root 1 (eigen value, λ = 12.7314), Pastinachidae are separated from the rest by their relatively longer ventral tail fold, whereas Himanturidae have no fin fold and the longest disc length. On root 2 (λ = 1.0282), Neotrygonidae are separated from the other families by their relatively large eye diameter. Specimens were all correctly predicted to their family in the classification matrix except for one individual of Dasyatidae classified as Neotrygonidae.

Dasyatidae
Type genus
*Dasyatis* Rafinesque, 1810, by original designation
Type species
*Dasyatis pastinaca* (Linnaeus, 1758)
Description

Body not wing-like, disc length slightly longer than width 101.0% (79.9–112.9%) DW, snout to cloaca length 87.9% (66.4–103.3%) DW, no denticles, thorns confined to midline of disc; tail whip-like, total length 277.2% (75.2–346.1%) DW, long but low ventral skin fold, ventral skin fold length 56.1% (31.7–99.3%) DW, tail slightly depressed at base, tail width 8.5% (3.7–12.3%) DW, tail height 5.0% (2.8–6.8%) DW, plain colour of either dark brown or black; subtriangular pelvic fin length 17.8% (11.1–23.1%) DW; eye diameter 3.8% (1.8–6.6%) DW; spiracle length 7.5% (5.1–10.4%) DW, interspiracular length 18.1% (12.5–22.1%) DW; distance between first pair of gill slits 21.4% (11.7–25.0%) DW, distance between fifth pair of gill slits 13.7% (8.3–16.0%) DW. Genera: *Dasyatis* and *Taeniurops*. *Taeniurops meyeni* has short tail, without body thorns.

**Neotrygonidae** (newly proposed)
Type genus
*Neotrygon* Castelnau, 1873, by original designation
Type species
*Neotrygon trigonoides* (Castelnau, 1873)
Description

Body not wing-like; disc length shorter than width 84.4% (79.2–87.3%) DW, snout to cloaca length 70.5% (68.2–73.4%) DW, no denticles, thorns confined to midline of disc; tail short, not whip-like, total length 197.3% (163.6–238.8%) DW, long but low ventral skin fold, ventral skin fold length 65.4% (54.8–77.6%) DW, tail slightly depressed at base, tail width 7.7% (6.6–8.2%) DW, tail height 5.2% (4.4–6.2%) DW, light colour or banded; subtriangular pelvic fin length 19.8% (14.2–22.4%) DW; eye diameter 5.5% (5.1–6.2%) DW; spiracle length 5.9% (5.2–6.7%) DW, interspiracular length 13.8% (12.7–15.4%) DW; distance between first pair of gill slits 16.4% (15.2–18.1%) DW, distance between fifth pair of gill slits 9.1% (8.5–10.2%) DW. Genera: *Neotrygon* and *Taeniura*. Most species have colourful spots on their body.

**Himanturidae** (newly proposed)
Type genus
*Himantura* Müller & Henle, 1837, by monotypy
Type species
*Himantura uarnak* (Gmelin, 1789)
Description

Body not wing-like; disc length slightly longer than width 104.1% (82.3–120.0%) DW, snout to cloaca length 88.5% (67.7–107.0%) DW, wide margin of granular or flat denticles, with or without thorns on midline, thorns granular (like pearl) or sharp if present; tail usually whip-like, total length 270.2% (154.7–468.0%) DW, without ventral skin fold, tail slightly depressed at base, tail width 9.2% (4.1–12.3%) DW, tail height 5.9% (3.4–9.0%) DW, plain in colour or with patterns; subtriangular pelvic fin length 16.1% (11.9–26.0%) DW; eye diameter 4.3% (1.0–6.7%) DW; spiracle length 6.9% (4.7–12.1%) DW, interspiracular length 18.5% (12.9–25.8%) DW; distance between first pair of gill slits 23.8% (14.6–30.8%) DW, distance between fifth pair of gill slits 15.8% (9.0–19.2%) DW. Genus: *Himantura*. Adult female *Himantura walga* however has short and bulbous tail.

**Pastinachidae** (newly proposed)
Type genus
*Pastinachus* Rüppell, 1829, by monotypy
Type species
*Pastinachus sephen* (Forsskål 1775)
Description

Body not wing-like; disc length slightly shorter than width 90.4% (82.6–97.6%) DW, snout to cloaca length 74.6% (68.9–78.7%) DW, wide margin of granular or flat denticles, pearl thorns on mid disc; tail whip-like, total length 338.5% (297.4–402.9%) DW, with long and large ventral skin fold, ventral skin fold length 102.7% (74.0–123.4%) DW, tail slightly depressed at base, tail width 10.9% (9.1–11.9%) DW, tail height 6.5% (5.7–7.2%) DW, plain in colour; subtriangular pelvic fin length 23.2% (20.5–26.9%) DW; eye diameter 2.6% (1.8–3.1%) DW; spiracle length 6.9% (5.9–7.7%) DW, interspiracular length 16.8% (14.3–18.7%) DW; distance between first pair of gill slits 19.8% (17.8–21.9%) DW, distance between fifth pair of gill slits 13.1% (12.2–13.8%) DW. Genus: *Pastinachus*.

### Key to the families of Order Myliobatiformes

**Fig 5:**
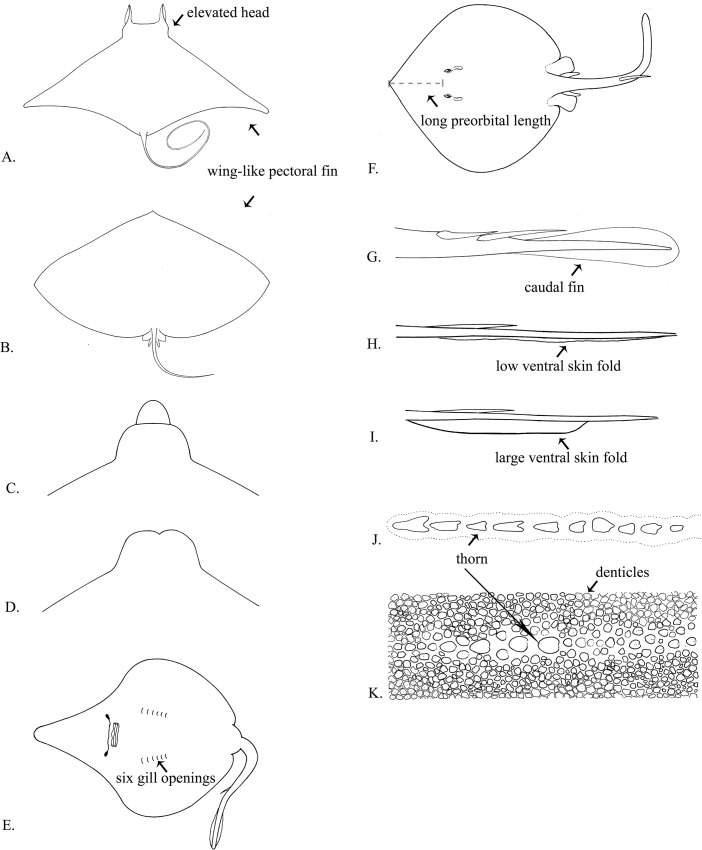
Morphological characters used in key to the families of Order Myliobatiformes. Wing-like body shape of Mobulidae (A) and Gymnuridae (B), Head of Myliobatidae (C), Head of Rhinopteridae (D), Gill openings of Hexatrigonidae (E), Plesiobatidae with long preorbital length (F), Short tail with caudal fin (G), Whip-like tail with low ventral skin fold (H), Whip-like tail with large ventral skin fold (I), Body thorns without denticles (J), and Body with thorns and denticles (K). Drawings adapted from photos and figures in Carpenter & Niem [1] and Last *et al*. [5].

**Fig 6:**
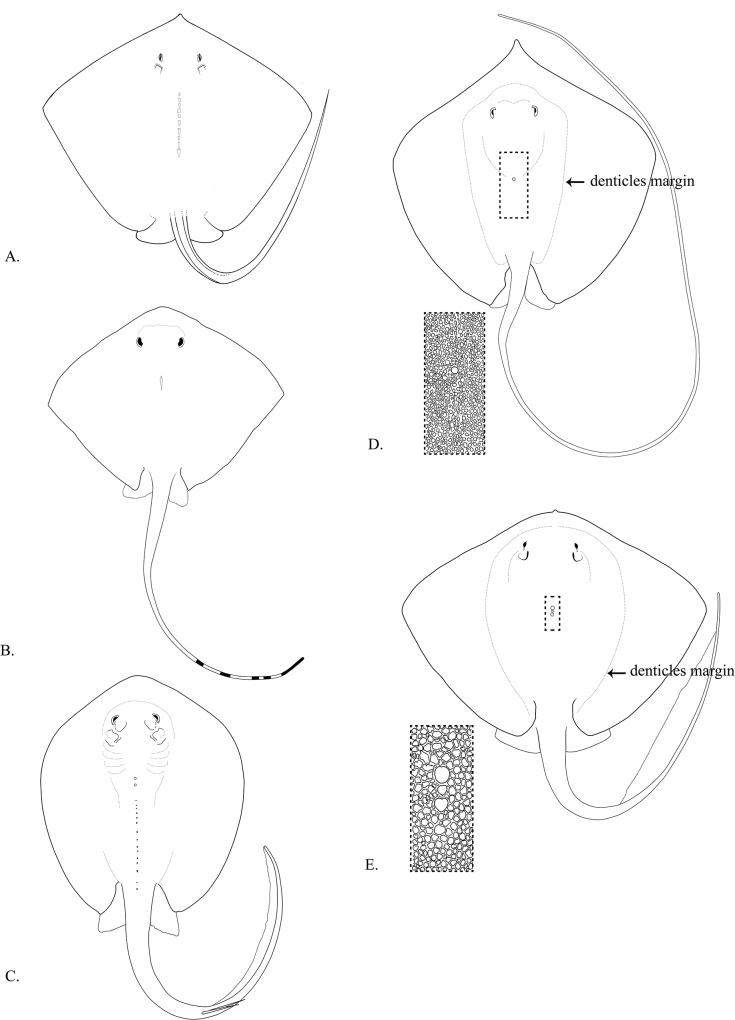
Dorsal surface of representative specimens of Dasyatidae (A), Neotrygonidae (*Neotrygon* species) (B), Neotrygonidae (*Taeniura* species) (C), Himanturidae (D) and Pastinachidae (E), showing thorns and/or denticle patterns on dorsal surface. Drawings adapted from photos in Carpenter & Niem [1] and Last *et al*. [5].

1a. Disc broad and laterally expanded with wing like pectoral fin, disc width less than 1.3 times disc length (Fig 5A & 5B)……2
1b. Disc not greatly expanded, diamond or round shaped, disc width more than 1.3 times disc length (Fig 6)……3
2a. Head not elevated, snout not differentiated into separate rostral or cephalic fins (Fig 5B)……**Gymnuridae**
2b. Head elevated, extended anterior to the pectoral fin with separate rostral fin or paired cephalic fins or horns (Fig 5A, 5C & 5D)……12
3a. Short and thick tail with well-developed caudal fin or with dorsal and ventral skin fold at rear end of tail, tail not whip like (Fig 5G)……4
3b. Caudal fin absent. Tail with or without ventral skin fold on midline of tail usually not reaching rear end of tail. Tail usually long and whip like (Fig 5H & 5I)……8
4a. Six pairs of gill openings with spiracles separated from the eyes (Fig 5E)……**Hexatrygonidae**
4b. Five pairs of gill openings with spiracles close to eyes……5
5a. Preorbital length of snout more than 6 times orbit diameter, disc surface with small granular denticles (Fig 5F)……**Plesiobatidae**
5b. Preorbital length of snout much lesser than 6 times orbit diameter, disc surface with or without denticles……6
6a. Disc surface with spiny or star like denticles over a wide margin, caudal fin reduced to dorsal and ventral skin flaps at rear end of tail. Body shape usually round or oval, non-angular at the side. Exclusively freshwater……**Potamotrygonidae**
6b. Disc surface smooth (rarely with spiny denticles), tail with well developed caudal fin. Body shape either round, oval or rhomboidal. Never found in freshwater……7
7a. Member of Eastern hemisphere (*Urolophus* and *Trygonoptera* species)……**Urolophidae**
7b. Member of Western hemisphere (*Urotrygon* and *Urobatis* species)......**Urotrygonidae**
8a. No prominent denticle. Low ventral skin fold (Fig 5H & 5J)……9
8b. Denticle band very broad (not observable in juvenile). Skin fold not as above (Fig 5K)......10
9a. Tail uniformly coloured, dorsal surface of disc uniform in colour, except *Taeniurops meyeni* (Fig 6A)……**Dasyatidae**
9b. Tail with either banded patterns or stripes, dorsal surface of disc with pattern of mostly spots (Fig 6B & 6C)……**Neotrygonidae** (proposed)
10a. Spiny denticles with no thorns on the body (not observable in juvenile), tail very broad based, tapering rapidly beyond sting to appear as 2 distinct portions, disc very broad (width more than 1.2 times length)……(*Dasyatis microps*)
10b. Granular or flat denticles, thorns on the body can be sharp or flat (not observable in juvenile), tail broad based or tapering evenly but not appearing as distinct portions, disc width less than 1.2 times length……11
11a. Tail with no ventral skin fold (Fig 6D)……**Himanturidae** (proposed)
11b. Tail with large ventral skin fold terminating well before tail tip (Fig 6E)……**Pastinachidae** (proposed)
12a. Snout in the form of prehensile, elongate, bilobate cephalic fins, laterally based on head (Fig 5A)……**Mobulidae**
12b. Snout in the form of single convex or low bilobate pair of rostral fins……13
13a. Rostral fin single and convex (Fig 5C)……**Myliobatidae**
13b. Rostral fin bilobate and broadly notched medially (Fig 5D)……**Rhinopteridae**

### Testing the functionality of the classification key for Myliobatiformes

The nine character states scored for the 17 test species of the current dasyatids formed the test character matrix in Table 4. None of these species were used to construct the family character matrix of the Myliobatiformes (Table 3) although some species were used to construct the phylogenetic trees. As observed, the test character matrix perfectly agrees with the derived character matrix for families of the Myliobatiformes; all 17 species were correctly identified to the four families.

**Table 4:** Character matrix for species within the four proposed families that were not included in the representative species list.

		Character states								
Family	Species	1	2	3	4	5	6	7	8	9
Himanturidae	*Himantura chaophraya*	1	2	1	3	1	1	0	0	0
	*Himantura dalyensis*	1	2	1	3	1	1	0	0	0
	*Himantura imbricata*	1	2	1	3	1	1	0	0	0
	*Himantura kittipongi*	1	2	1	3	1	1	0	0	0
	*Himantura lobistoma*	1	2	1	3	1	1	0	0	0
	*Himantura oxyrhyncha*	1	2	1	3	1	1	1	0	0
	*Himantura signifier*	1	2	1	3	1	1	0	0	0
Pastinachidae	*Pastinachus stellurostris*	1	2	1	3	1	1	0	2	0
Dasyatidae	*Dasyatis brevicaudata*	1	1	1	3	1	1	0	1	0
	*Dasyatis centroura*	1	1	1	3	1	1	0	1	0
	*Dasyatis fluviorum*	1	1	1	3	1	1	0	1	0
	*Dasyatis parvonigra*	1	1	1	3	1	1	0	1	0
	*Dasyatis thetidis*	1	1	1	3	1	1	0	1	0
	*Dasyatis ushiei*	1	1	1	3	1	1	0	1	0
	*Taeniurops grabata*	1	1	1	3	1	0	0	1	0
Neotrygonidae	*Neotrygon ningalooensis*	1	1	1	3	1	0	1	1	0
	*Neotrygon picta*	1	1	1	3	1	0	1	1	0

See Table 3 for detailed explanation on the differentiation of the morphological characters used.

## Discussion

The COI, ND2 and RAG1 genes that were used in the present study had revealed the non-monophyletic nature of the present Dasyatidae. All genes are successfully used in the present study to resolve the specific relationships of the problematic current Dasyatidae and the familial relationships of the Myliobatiformes. Neither of these genes nor other available genes has ever been studied at the family level in elasmobranchs. Our study using additional morphological information has erected a natural classification key for the Myliobatiformes by removing previously used characters the cause of *incertae sedis* and past misclassifications.

The results of our study agree with Cerutti-Pereyra *et al*. [10] study using COI gene showing clear taxonomic classification in Myliobatiformes with four major clusters in Dasyatidae. As shown in the phylogenetic tree, sequences of samples belonging to the same species formed the smallest clusters at the distal end of the trees, e.g. *Dasyatis zugei*, *Neotrygon kuhlii*, *Himantura pastinacoides*, *Pastinachus atrus* and similarly for sequences of the same genus. The clusters and their subclusters shown in the phylogenetic trees of COI, ND2 and RAG1 genes were supported by the uncorrected p-distance with the smallest intraspecific distance (0 to 4.91% in COI and 0 to 3.66% in ND2). For RAG1 gene, the currently available sequences were insufficient to present conclusive result at intraspecific level. However, based on the present available data, the uncorrected p-distances ranged from 0 to 1.28%.

At the genus level, the uncorrected p-distance was higher than that of species level i.e. 1.87 to 18.46% for COI, 4.53 to 19.86% for ND2 and 0 to 4.81% for RAG1. For COI gene, the mean distance at genus level in the present study (12.03%) was found to be higher than that of Cerutti-Pereyra *et al*. [10] (8.85%), Ward *et al*. [33] (9.93%) and Ward *et al*. [7] (7.48%) but similar to Zhang & Hanner [34] (13.55%). In terms of the distance variability for COI gene, the variation in the present study (16.59%) is higher than that of Cerutti-Pereyra *et al*. [10] (10.6%) but lower than that of Ward *et al*. [33] (20.63%), Ward *et al*. [7] (24.18%) and Zhang & Hanner [34] (25.35%). For ND2 gene, the mean distance at genus level in the present study (mean 14.67%) is higher than that of Naylor *et al*. [11] (mean 10.16%) but the distance variation in the present study (15.33%) is lower than that of Naylor *et al*. [11] (26.98%). For the RAG1 gene, there was no available reference on p-distance at the species or genus level of batoids.

The mean distance at the genus level is higher in the present study as compared to those reported by others, but still within the reported range. Although the range of p-distance at the genus level overlapped with that at the family level (18.63%, 21.53% and 5.97% for COI, ND2 and RAG1 genes respectively) (see Table 1 & 2), the mean p-distance at the family level was significantly higher than at the genus level. This confirmed the functionality of the used genes in elucidating the taxonomic classification at family level.

According to Carpenter & Niem [1] and Last *et al*. [5], the species of *Himantura*, *Pastinachus*, *Dasyatis* (with *Taeniurops meyeni*), *Neotrygon* and *Taeniura* belonged to the Dasyatidae. However, McEachran & Aschliman [2] and Aschliman *et al*. [4] suggested that their examined species of *Dasyatis* and *Neotrygon* in the Dasyatidae were not monophyletic. Both Cerutti-Pereyra *et al*. [10] and our study further confirm non-monophyly. Our study shows that the *Himantura* and *Pastinachus* species are also not monophyletic if placed within the current Dasyatidae (see Figs 1 & 2). The p-distances between the species clusters (families) of studied genes are clearly large thus substantiating the four distinct clades within the current Dasyatidae. The results suggest taxonomic separation at the family level. Here, we proposed three new families, namely, Neotrygonidae (to include *Neotrygon* and *Taeniura* spp.), Pastinachidae (*Pastinachus* spp.) and the Himanturidae (*Himantura* spp.), while retaining the Dasyatidae which include the *Dasyatis* and *Taeniurops* species. The proposed elevation of these three clusters to family level is appropriate since elevation will maintain their monophyletic relationship. Discriminant analysis of their character morphometrics further shows their distinctness (see Fig 4). The single member, *Dasyatis microps*, with available COI and ND2 genes sequence in GenBank, oddly did not group into the Dasyatis clade and possessed a unique character set that could not fit into any of the other families (see Table 3); it may suggest a misidentification that could belong to a new family. The present study supports the change in name of both *Taeniura meyeni* and *Taeniura grabata* to *Taeniurops meyeni* and *Taeniurops grabata*, respectively [4, 20], and their retention within the Dasyatidae.

The usefulness of both morphology and molecular information to arrive at a natural classification system for the stingrays has never been employed in previous works [2, 7, 9–11, 35–37]. Naylor *et al*. [35] focused on classification at the ordinal level by comparing their constructed molecular trees with the available morphological trees of others, but did not combine their usefulness. However, the use of combined morphological and molecular information in taxonomy is not new, being applied to plants [38, 39] and arthropods [40, 41], although morphological information only contributed to about 5% of the used characters in the phylogenetic tree [38, 39]. Ruhfel *et al*. [39] working on fossil plants further concluded that the topology from molecular data alone was better than the combination of both morphology and molecular data. As suggested by Ruhfel *et al*. [39], the possible reason that morphological traits showed weak contribution to phylogenetic classification is the lack of better morphological data that clearly separate the clades. However, the approach we used in the present study, i.e. by inserting the morphological characters into the constructed phylogenetic tree, ensures that the suite of contrasting morphological traits is compatible to the molecular classification.

## Conclusions

Molecular genetics successfully elucidated the phylogenetic relationships of the Dasyatidae stingrays, and suggests that the current family is non-monophyletic and should be split into four families, including itself with three new families, Neotrygonidae, Himanturidae and Pastinachidae. By resolving the non-monophyletic problem, the use of a suite of nine character states enables the natural classification of the Myliobatiformes into thirteen families based on morphology.

## Supporting Information

S1 Table Specimen collection details for all sequences obtained in this study. (DOCX)

S2 Table Checklist of analysed species used for molecular markers, character matrix, test subjects and morphometrics. All samples are obtained from GeneBank or published references, except those in bold (new samples).
(DOCX)

S3 Table Character matrix of representative species within Myliobatiformes (present study). See Table 3 for detailed explanation on the differentiation of the morphological characters used.
(DOCX)

S4 Table Morphometric measurements of Himanturidae, Dasyatidae, Neotrygonidae and Pastinachidae. Measurements are expressed as percentage of disc width.
(DOCX)
