# Correction: Molecular and Morphological Analyses Reveal Phylogenetic Relationships of Stingrays Focusing on the Family Dasyatidae (Myliobatiformes)

**DOI:** 10.1371/journal.pone.0129411

**Published:** 2015-05-26

**Authors:** Kean Chong Lim, Phaik-Eem Lim, Ving Ching Chong, Kar-Hoe Loh

There are errors in identification steps 1a and 1b in the “Key to the families of Order Myliobatiformes” subsection of the Results. The correct step 1a is: Disc broad and laterally expanded with wing like pectoral fin, disc width more than 1.3 times disc length (Fig 5A & 5B)……2. The correct step 1b is: Disc not greatly expanded, diamond or round shaped, disc width less than 1.3 times disc length (Fig 6)……3.
