# Supplementary material for: Molecular and Morphological Analyses Reveal Phylogenetic Relationships of Stingrays Focusing on the Family Dasyatidae (Myliobatiformes)
Source: PLoS One. 2015 Apr 13;10(4):e0120518. doi: 10.1371/journal.pone.0120518 (PMC4395009; doi:10.1371/journal.pone.0120518)
Supplement: S1 Table — (DOCX) [file pone.0120518.s001.docx]

**Table S1.** Specimen collection details for all sequences obtained in this study.

| No. | Species | Laboratory code | Tree code | Gene | Location | Collection Date | Accession No. |
| --- | --- | --- | --- | --- | --- | --- | --- |
| 1 | *Pastinachus atrus* | 1_PATR2_CO1 | *Pastinachus atrus* 2 | COI | Malaysia: Tawau | 13-May-2013 | KM072986 |
| 2 | *Pastinachus atrus* | 1_PATR3_CO1 | *Pastinachus atrus* 3 | COI | Malaysia: Semporna | 11-May-2013 | KM072987 |
| 3 | *Pastinachus atrus* | 1_PATR1_CO1 | *Pastinachus atrus* 1 | COI | Malaysia: Sandakan | 10-Mar-2013 | KM072988 |
| 4 | *Pastinachus gracilicaudus* | 1_PGRA2_CO1 | *Pastinachus gracilicaudus* 2 | COI | Malaysia: Sandakan | 11-Mar-2013 | KM072989 |
| 5 | *Pastinachus gracilicaudus* | 1_PGRA1_CO1 | *Pastinachus gracilicaudus* 1 | COI | Malaysia: Sandakan | 7-Mar-2013 | KM072990 |
| 6 | *Himantura jenkensii* | 1_HJEN3_CO1 | *Himantura jenkensii* 3 | COI | Malaysia: Semporna | 9-Mar-2013 | KM072991 |
| 7 | *Himantura jenkensii* | 1_HJEN2_CO1 | *Himantura jenkensii* 2 | COI | Malaysia: Semporna | 11-May-2013 | KM072992 |
| 8 | *Himantura jenkensii* | 1_HJEN1_CO1 | *Himantura jenkensii* 1 | COI | Malaysia: Sandakan | 11-Mar-2013 | KM072993 |
| 9 | *Himantura walga* | 1_HWAL2a_CO1 | *Himantura walga* 2 | COI | Malaysia: Kuala Selangor | 5-Jul-2012 | KM072994 |
| 10 | *Himantura walga* | 1_HWAL1_CO1 | *Himantura walga* 1 | COI | Malaysia: Kuala Selangor | 3-Sep-2012 | KM072995 |
| 11 | *Himantura leoparda* | 1_HLEO2_CO1 | *Himantura leoparda* 2 | COI | Malaysia: Sandakan | 10-Mar-2013 | KM072996 |
| 12 | *Himantura leoparda* | 1_HLEO1_CO1 | *Himantura leoparda* 1 | COI | Malaysia: Sandakan | 8-Mar-2013 | KM072997 |
| 13 | *Himantura leoparda* | 1_HLEO3_CO1 | *Himantura leoparda* 3 | COI | Malaysia: Sandakan | 12-Mar-2013 | KM072998 |
| 14 | *Himantura uarnak* | 1_HUAR3_CO1 | *Himantura uarnak* 3 | COI | Malaysia: Semporna | 11-May-2013 | KM072999 |
| 15 | *Himantura uarnak* | 1_HUAR1_CO1 | *Himantura uarnak* 1 | COI | Malaysia: Sandakan | 11-Mar-2013 | KM073000 |
| 16 | *Himantura undulata* | 1_HUND1_CO1 | *Himantura undulata* | COI | Malaysia: Tawau | 12-May-2013 | KM073001 |
| 17 | *Himantura gerrardi* | 1_HGER1_CO1 | *Himantura gerrardi* 1 | COI | Malaysia: Sandakan | 7-Mar-2013 | KM073002 |
| 18 | *Himantura gerrardi* | 1_HGER3_CO1 | *Himantura gerrardi* 3 | COI | Malaysia: Tawau | 13-May-2013 | KM073003 |
| 19 | *Himantura pastinacoides* | 1_HPAS1_CO1 | *Himantura pastinacoides* 1 | COI | Malaysia: Kuala Selangor | 30-Nov-2013 | KM073004 |
| 20 | *Himantura pastinacoides* | 1_HPAS3_CO1 | *Himantura pastinacoides* 3 | COI | Malaysia: Sandakan | 12-Mar-2013 | KM073005 |
| 21 | *Himantura pastinacoides* | 1_HPAS2_CO1 | *Himantura pastinacoides* 2 | COI | Malaysia: Sandakan | 8-Mar-2013 | KM073006 |
| 22 | *Himantura uarnacoides* | 1_HUAC3_CO1 | *Himantura uarnacoides* 3 | COI | Malaysia: Sandakan | 8-Mar-2013 | KM073007 |
| 23 | *Himantura uarnacoides* | 1_HUAC1_CO1 | *Himantura uarnacoides* 1 | COI | Malaysia: Sandakan | 7-Mar-2013 | KM073008 |
| 24 | *Himantura uarnacoides* | 1_HUAC2_CO1 | *Himantura uarnacoides* 2 | COI | Malaysia: Sandakan | 8-Mar-2013 | KM073009 |
| 25 | *Himantura fai* | 1_HFAI2_CO1 | *Himantura fai* | COI | Malaysia: Semporna | 11-May-2013 | KM073010 |
| 26 | *Mobula kuhlii* | 1_MKUH1_CO1 | *Mobula kuhlii* | COI | Malaysia: Sandakan | 12-Mar-2013 | KM073011 |
| 27 | *Mobula thurstoni* | 1_MTHU1_CO1 | *Mobula thurstoni* | COI | Malaysia: Semporna | 10-May-2013 | KM073012 |
| 28 | *Rhinoptera javanica* | 1_RJAV1a_CO1 | *Rhinoptera javanica* 1 | COI | Malaysia: Sandakan | 8-Mar-2013 | KM073013 |
| 29 | *Rhinoptera javanica* | 1_RJAV2_CO1 | *Rhinoptera javanica* 2 | COI | Malaysia: Sandakan | 10-Mar-2013 | KM073014 |
| 30 | *Rhinoptera jayakari* | 1_RJAY2_CO1 | *Rhinoptera jayakari* | COI | Malaysia: Sandakan | 13-Mar-2013 | KM073015 |
| 31 | *Dasyatis bennetti* | 1_DBEN2_CO1 | *Dasyatis bennetti* 2 | COI | Malaysia: Kuala Selangor | 5-Jul-2012 | KM073016 |
| 32 | *Dasyatis bennetti* | 1_DBEN1_CO1 | *Dasyatis bennetti* 1 | COI | Malaysia: Kuala Selangor | 14-Nov-2012 | KM073017 |
| 33 | *Dasyatis bennetti* | 1_DBEN3_CO1 | *Dasyatis bennetti* 3 | COI | Malaysia: Kuala Selangor | 3-Sep-2012 | KM073018 |
| 34 | *Taeniurops meyeni* | 1_TMEY1_CO1 | *Taeniurops meyeni* | COI | Malaysia: Sandakan | 8-Mar-2013 | KM073019 |
| 35 | *Dasyatis zugei* | 1_DZUG1_CO1 | *Dasyatis zugei* 1 | COI | Malaysia: Kuala Selangor | 18-Oct-2012 | KM073020 |
| 36 | *Dasyatis zugei* | 1_DZUG3_CO1 | *Dasyatis zugei* 3 | COI | Malaysia: Kuala Selangor | 18-Oct-2012 | KM073021 |
| 37 | *Dasyatis zugei* | 1_DZUG2_CO1 | *Dasyatis zugei* 2 | COI | Malaysia: Kuala Selangor | 5-Jul-2012 | KM073022 |
| 38 | *Neotrygon kuhlii* | 1_NKUH1_CO1 | *Neotrygon kuhlii* 1 | COI | Malaysia: Kuala Selangor | 3-Oct-2012 | KM073023 |
| 39 | *Neotrygon kuhlii* | 1_NKUH2_CO1 | *Neotrygon kuhlii* 2 | COI | Malaysia: Sandakan | 12-Mar-2013 | KM073024 |
| 40 | *Neotrygon kuhlii* | 1_NKUH3_CO1 | *Neotrygon kuhlii* 3 | COI | Malaysia: Sandakan | 7-Mar-2013 | KM073025 |
| 41 | *Taeniura lymma* | 1_TLYM2_CO1 | *Taeniura lymma* 2 | COI | Malaysia: Sandakan | 7-Mar-2013 | KM073026 |
| 42 | *Taeniura lymma* | 1_TLYM3_CO1 | *Taeniura lymma* 3 | COI | Malaysia: Sandakan | 13-Mar-2013 | KM073027 |
| 43 | *Aetobatus ocellatus* | 1_AOCE1_CO1 | *Aetobatus ocellatus* 1 | COI | Malaysia: Sandakan | 8-Mar-2013 | KM073028 |
| 44 | *Aetobatus ocellatus* | 1_AOCE2_CO1 | *Aetobatus ocellatus* 2 | COI | Malaysia: Sandakan | 10-Mar-2013 | KM073029 |
| 45 | *Gymnura zonura* | 1_GZON1_CO1 | *Gymnura zonura* 1 | COI | Malaysia: Sandakan | 9-Mar-2013 | KM073030 |
| 46 | *Gymnura zonura* | 1_GZON2_CO1 | *Gymnura zonura* 2 | COI | Malaysia: Tawau | 12-May-2013 | KM073031 |
| 47 | *Gymnura zonura* | 1_GZON3_CO1 | *Gymnura zonura* 3 | COI | Malaysia: Tawau | 12-May-2013 | KM073032 |
| 48 | *Pastinachus atrus* | 1_Patr_1 | *Pastinachus atrus* 1 | ND2 | Malaysia: Sandakan | 10-Mar-2013 | KM073033 |
| 49 | *Pastinachus gracilicaudus* | 1_Pgra_2 | *Pastinachus gracilicaudus* 2 | ND2 | Malaysia: Sandakan | 11-Mar-2013 | KM073034 |
| 50 | *Neotrygon kuhlii* | 1_Nkuh_3 | *Neotrygon kuhlii* 3 | ND2 | Malaysia: Sandakan | 7-Mar-2013 | KM073035 |
| 51 | *Neotrygon kuhlii* | 2_Nkuh_2 | *Neotrygon kuhlii* 2 | ND2 | Malaysia: Sandakan | 12-Mar-2013 | KM073036 |
| 52 | *Taeniura lymma* | 1_Tlym_2 | *Taeniura lymma* 2 | ND2 | Malaysia: Sandakan | 7-Mar-2013 | KM073037 |
| 53 | *Taeniura lymma* | 1_Tlym_3 | *Taeniura lymma* 3 | ND2 | Malaysia: Sandakan | 13-Mar-2013 | KM073038 |
| 54 | *Dasyatis bennetti* | 1_Dben_1 | *Dasyatis bennetti* 1 | ND2 | Malaysia: Kuala Selangor | 14-Nov-2012 | KM073039 |
| 55 | *Dasyatis bennetti* | 1_Dben_2 | *Dasyatis bennetti* 2 | ND2 | Malaysia: Kuala Selangor | 5-Jul-2012 | KM073040 |
| 56 | *Dasyatis zugei* | 1_Dzug_2 | *Dasyatis zugei* 2 | ND2 | Malaysia: Kuala Selangor | 5-Jul-2012 | KM073041 |
| 57 | *Dasyatis zugei* | 1_Dzug_1 | *Dasyatis zugei* 1 | ND2 | Malaysia: Kuala Selangor | 18-Oct-2012 | KM073042 |
| 58 | *Himantura jenkensii* | 1_Hjen_3 | *Himantura jenkensii* 3 | ND2 | Malaysia: Sandakan | 9-Mar-2013 | KM073043 |
| 59 | *Himantura leoparda* | 1_Hleo_2 | *Himantura leoparda* 2 | ND2 | Malaysia: Sandakan | 10-Mar-2013 | KM073044 |
| 60 | *Himantura leoparda* | 1_Hleo_1 | *Himantura leoparda* 1 | ND2 | Malaysia: Sandakan | 8-Mar-2013 | KM073045 |
