# Supplementary material for: Molecular and Morphological Analyses Reveal Phylogenetic Relationships of Stingrays Focusing on the Family Dasyatidae (Myliobatiformes)
Source: PLoS One. 2015 Apr 13;10(4):e0120518. doi: 10.1371/journal.pone.0120518 (PMC4395009; doi:10.1371/journal.pone.0120518)
Supplement: S2 Table — All samples are obtained from GeneBank or published references, except those in bold (new samples). (DOCX) [file pone.0120518.s002.docx]

**Table S2.** Checklist of analysed species used for molecular markers, character matrix, test subjects and morphometrics. All samples are obtained from GeneBank or published references, except those in bold (new samples).

| Family | Species | COI | ND2 | RAG1 | Character matrix | Test | Morphometrics |
| --- | --- | --- | --- | --- | --- | --- | --- |
| Dasyatidae | *Dasyatis akajei* | AB485681, EU339356, JF952715 |  | KC249805 | √ |  |  |
|  | *Dasyatis americana* |  |  | JN184237 |  |  |  |
|  | *Dasyatis bennetti* | JN242685, JQ681485, FJ237982, **KM073016, KM073017, KM073018** | **KM073039, KM073040** |  | √ |  | 65 |
|  | *Dasyatis brevicaudata* |  | JQ519028 |  |  | √ |  |
|  | *Dasyatis brevis* |  |  | JN184114 |  |  |  |
|  | *Dasyatis centroura* |  |  |  |  | √ |  |
|  | *Dasyatis fluviorum* | DQ108183, JQ765505 |  |  |  | √ |  |
|  | *Dasyatis microps* | KJ749659, KJ749660, EU541310 | JQ518779 |  | √ |  |  |
|  | *Dasyatis parvonigra* |  |  |  |  | 5 | 4 |
|  | *Dasyatis thetidis* |  |  |  |  | √ |  |
|  | *Dasyatis ushiei* |  | JQ518780 | KC249806 |  | √ |  |
|  | *Dasyatis zugei* | EU398762, JQ681524, **KM073020, KM073021, KM073022** | JN184289, JQ518790, **KM073041, KM073042** | JN184238 | √ |  | 54 |
|  | *Taeniurops grabata** |  |  | JN184241 |  | √ |  |
|  | *Taeniurops meyeni** | HM467797, JQ765555, **KM073019** | JN184293, JQ518822 | KC249811, JN184242 | √ |  |  |
| Himanturidae | *Himantura astra* | EU398866, EU398867 |  |  |  |  | 15 |
| (proposed) | *Himantura chaophraya* |  | JQ518804 |  |  | √ | 2 |
|  | *Himantura dalyensis* |  |  |  |  | 9 | 4 |
|  | *Himantura fai* | JQ431866, JF493647, **KM073010** |  |  | √ |  |  |
|  | *Himantura fava* | DQ108167 |  |  |  |  |  |
|  | *Himantura gerrardi* | JX263424, JF493650, **KM073002, KM073003** |  | JN184252 | √ |  |  |
|  | *Himantura granulata* | JQ765514 |  |  | √ |  |  |
|  | *Himantura imbricata* |  |  |  |  | √ |  |
|  | *Himantura jenkensii* | EU398850, DQ108169, **KM072991, KM072992, KM072993** | JQ518810, **KM073043** |  | √ |  |  |
|  | *Himantura kittipongi* |  |  |  |  | √ | 5 |
|  | *Himantura krempfi* |  |  |  |  |  | 3 |
|  | *Himantura leoparda* | JX263335, JQ929047, JF493652, JX263418, **KM072996, KM072997, KM072998** | JQ518802, **KM073044, KM073045** |  |  |  | 8 |
|  | *Himantura lobistoma* |  |  | JN184253 |  | √ | 6 |
|  | *Himantura oxyrhyncha* |  |  |  |  | √ | 2 |
|  | *Himantura pastinacoides* | EU398854, EU398857, **KM073004, KM073005, KM073006** | JQ518797 |  | √ |  | 1 |
|  | *Himantura schmardae* |  |  | JN184126 |  |  |  |
|  | *Himantura signifier* |  | JQ519072 |  |  | √ | 7 |
|  | *Himantura toshi* | EU398868, EU398869 |  |  | √ |  |  |
|  | *Himantura uarnacoides* | DQ108166, EU398871, **KM073007, KM073008, KM073009** | JN184306, JQ519086 | JN184254 | √ |  |  |
|  | *Himantura uarnak* | JX263360, **KM072999, KM073000** | JQ518800, JQ518805, JQ518807 | JN184255 | √ |  |  |
|  | *Himantura undulata* | JX263336, **KM073001** |  |  |  |  |  |
|  | *Himantura walga* | EU398874, EU398876, **KM072994, KM072995** | JN184308, JQ518798 | KC249807, JN184256 | √ |  | 52 |
| Neotrygonidae | *Neotrygon annotata* | EU398728, EU398731 |  | KC249769, KC249768, KC249771, KC249770 | √ |  |  |
| (proposed) | *Neotrygon kuhlii* | KC249905, AB485685, **KM073023, KM073024, KM073025** | JQ519098, JQ519093, JQ518811, **KM073035, KM073036** | KC249797, JN184115, KC248793 | √ |  | 1 |
|  | *Neotrygon leylandi* | EU398747, JQ765538, EU398751 |  | KC249798, KC249799 | √ |  | 3 |
|  | *Neotrygon ningalooensis* |  |  | KC249804, KC249802, KC249803 |  | √ |  |
|  | *Neotrygon picta* |  |  | KC249800, KC249801 |  | 13 | 6 |
|  | *Neotrygon trigonoides* | JX263420 |  |  |  |  |  |
|  | *Taeniura lymma* | FJ584170, JQ929048, JQ765551, **KM073026, KM073027** | JQ518820, JQ518819, **KM073037, KM073038** | KC249809, JN184117, KN249810 | √ |  |  |
| Pastinachidae | *Pastinachus atrus* | DQ108161, EU398971, **KM072986, KM072987, KM072988** | JN184290, JQ518815, **KM073033** | KC249808, JN184239 | √ |  |  |
| (proposed) | *Pastinachus gracilicaudus* | DQ108160, EU398968, **KM072989, KM072990** | JQ519060, **KM073034** |  | √ |  | 15 |
|  | *Pastinachus solocirostris* | EF609431, EU398977 |  | JN184116 | √ |  |  |
|  | *Pastinachus stellurostris* |  |  |  |  | √ | 4 |
| Gymnuridae | *Gymnura cf poecilura* |  | JQ518834, JQ519068 |  | √ |  |  |
|  | *Gymnura crebripunctata* |  |  | JN184119 |  |  |  |
|  | *Gymnura micrura* |  |  | JN184244 |  |  |  |
|  | *Gymnura* (*Aetoplatae*) *zonura* | EU398808, EU308809, **KM073030, KM073031, KM073032** |  | JN184243 | √ |  |  |
| Myliobatidae | *Aetobatus flagellum* | EU339362, FJ812205 | JQ518839 |  | √ |  |  |
|  | *Aetobatus ocellatus* (narinari) | JX978339, JF492797, EU398507, EU398508, **KM073028, KM073029** | JQ518988, JQ519092 | JN184121 | √ |  |  |
|  | *Aetomylaeus maculatus* |  |  | JN184245 | √ |  |  |
|  | *Aetomylaeus nichofii* |  |  | JN184246 | √ |  |  |
|  | *Aetomylaeus vespertilio* |  |  | JN184247 |  |  |  |
|  | *Myliobatis califormica* |  |  | JN184249, JN184123 |  |  |  |
|  | *Myliobatis freminvillii* |  |  | JN184250 |  |  |  |
| Mobulidae | *Manta birostris* |  |  | JN184248 |  |  |  |
|  | *Mobula japanica* | EU398908, EU398909 | JQ519163 | JN184122 | √ |  |  |
|  | *Mobula kuhlii* | EU398907, HQ589279, JF493899, **KM073011** | JQ518836 |  | √ |  |  |
|  | *Mobula tarapacana* | EU398912, EU398913 |  |  | √ |  |  |
|  | *Mobula thurstoni* | EU398917, EU398918, **KM073012** | JN184300, JQ519161 | JN184173 | √ |  |  |
| Rhinopteridae | *Rhinoptera javanica* | DQ108133, GU805122, JF494383, **KM073013, KM073014** | JQ518924 |  | √ |  |  |
|  | *Rhinoptera jayakari* | **KM073015** | JQ518917 |  | √ |  |  |
|  | *Rhinoptera steindachneri* |  |  | JN184124 |  |  |  |
| Plesiobatidae | *Plesiobatis daviesi* | EU398978, HM467801 | KF927936, KF927935, JQ519131 |  | √ |  |  |
| Hexatrygonidae | *Hexatrygon bickelli* |  | JQ518835 |  | √ |  |  |
| Potamotrygonidae | *Potamotrygon motoro* | JN989160, JN989159 |  |  | √ |  |  |
|  | *Potamotrygon falkneri* | JN989146, JN989144 |  |  | √ |  |  |
|  | *Potamotrygon tatianae* |  |  |  | 7 |  |  |
| Urolophidae | *Urolophus kapalensis* | EU399120, EF609491 |  |  |  |  |  |
|  | *Urolophus sufflavus* | EU399134, EF609492 |  |  |  |  |  |
|  | *Urolophus paucimaculatus* |  | JN184311, JQ518938 |  | √ |  |  |
|  | *Urolophus cruciatus* |  |  |  | √ |  |  |
|  | *Trygonoptera testacea* | EU399089, EU399085 | JN184310, JQ518935 |  | √ |  |  |
|  | *Trygonoptera imitata* |  |  |  | 19 |  |  |
| Urotrygonidae | *Urobatis jamaicensis* | KF930520, GU225505, GU225504 | JQ518941 |  | √ |  |  |
|  | *Urobatis halleri* |  |  |  | √ |  |  |
|  | *Urobatis venezuelae* |  |  |  | √ |  |  |
|  | *Urobatis maculatus* |  |  |  | √ |  |  |
|  | *Urotrygon concentricus* |  | JQ519167 |  | √ |  |  |
|  | *Urotrygon rogersi* |  | JQ519162 |  |  |  |  |
| Carcharhinidae | *Carcharhinusamblyrhynchos* | EF609308 | JQ519095 |  |  |  |  |
|  | *Carcharhinusplumbeus* | EU398639 | JQ518632 | AY462152 |  |  |  |
| Total samples in analysis | | 138 | 62 | 49 |  |  |  |
| Total recorded Myliobatiformes | | 220 | 220 | 220 | 220 |  |  |
| Total recorded Dasyatidae | | 88 | 88 | 88 | 88 | 88 | 88 |
| Percentage of Myliobatiformes used in present study | | 20% (43 sp) | 15% (33 sp) | 15% (34 sp) | 21% (47 sp) |  |  |
| Percentage of Dasyatidae used in present study | | 31% (27sp) | 20% (18 sp) | 24% (21 sp) | 24% (21 sp) | 19% (17sp) | 22% (19sp) |

** formerly Taeniura meyeni* and *Taeniura grabata,* see Aschliman *et al.* [4] and Last, Steven [20]. Numerals indicate sample size of species used in the morphological analysis whereas ticks indicate unknown sample size.
