# Supplementary material for: Molecular and Morphological Analyses Reveal Phylogenetic Relationships of Stingrays Focusing on the Family Dasyatidae (Myliobatiformes)
Source: PLoS One. 2015 Apr 13;10(4):e0120518. doi: 10.1371/journal.pone.0120518 (PMC4395009; doi:10.1371/journal.pone.0120518)
Supplement: S3 Table — See Table 3 for detailed explanation on the differentiation of the morphological characters used. (DOCX) [file pone.0120518.s003.docx]

**Table S3.** Character matrix of representative species within Myliobatiformes (present study). See Table 3 for detailed explanation on the differentiation of the morphological characters used.

|  | Character states | | | | | | | | |
| --- | --- | --- | --- | --- | --- | --- | --- | --- | --- |
| Species | 1 | 2 | 3 | 4 | 5 | 6 | 7 | 8 | 9 |
| *Mobula kuhii* | 0 | 0 | 0 | 2 | 1 | 1 | 0 | 0 | 0 |
| *Mobula thurstoni* | 0 | 0 | 0 | 2 | 1 | 1 | 0 | 0 | 0 |
| *Mobula japanica* | 0 | 0 | 0 | 2 | 1 | 1 | 0 | 0 | 0 |
| *Mobula tarapacana* | 0 | 0 | 0 | 2 | 1 | 1 | 0 | 0 | 0 |
| *Rhinoptera javanica* | 0 | 0 | 0 | 1 | 1 | 1 | 0 | 0 | 0 |
| *Rhinoptera jayakari* | 0 | 0 | 0 | 1 | 1 | 1 | 0 | 0 | 0 |
| *Aetobatus ocellatus (narinari)* | 0 | 0 | 0 | 0 | 1 | 1 | 0 | 0 | 0 |
| *Aetobatus flagellum* | 0 | 0 | 0 | 0 | 1 | 1 | 0 | 0 | 0 |
| *Aetomylaeus maculatus* | 0 | 0 | 0 | 0 | 1 | 1 | 0 | 0 | 0 |
| *Aetomylaeus nichofii* | 0 | 0 | 0 | 0 | 1 | 1 | 0 | 0 | 0 |
| *Gymnura zonura* | 0 | 0 | 1 | 3 | 1 | 1 | 1 | 0 | 0 |
| *Gymnura poecilura* | 0 | 0 | 1 | 3 | 1 | 1 | 1 | 0 | 0 |
| *Himantura jenkensii* | 1 | 2 | 1 | 3 | 1 | 1 | 0 | 0 | 0 |
| *Himantura gerrardi* | 1 | 2 | 1 | 3 | 1 | 1 | 1 | 0 | 0 |
| *Himantura pastinacoides* | 1 | 2 | 1 | 3 | 1 | 1 | 0 | 0 | 0 |
| *Himantura toshi* | 1 | 2 | 1 | 3 | 1 | 1 | 1 | 0 | 0 |
| *Himantura uarnacoides* | 1 | 2 | 1 | 3 | 1 | 1 | 0 | 0 | 0 |
| *Himantura walga* | 1 | 2 | 1 | 3 | 1 | 0 | 0 | 0 | 0 |
| *Himantura fai* | 1 | 2 | 1 | 3 | 1 | 1 | 0 | 0 | 0 |
| *Himantura granulata* | 1 | 2 | 1 | 3 | 1 | 1 | 0 | 0 | 0 |
| *Himantura uarnak* | 1 | 2 | 1 | 3 | 1 | 1 | 1 | 0 | 0 |
| *Pastinachus atrus* | 1 | 2 | 1 | 3 | 1 | 1 | 0 | 2 | 0 |
| *Pastinachus gracilicaudus* | 1 | 2 | 1 | 3 | 1 | 1 | 0 | 2 | 0 |
| *Pastinachus solocirostris* | 1 | 2 | 1 | 3 | 1 | 1 | 0 | 2 | 0 |
| *Dasyatis bennetti* | 1 | 1 | 1 | 3 | 1 | 1 | 0 | 1 | 0 |
| *Dasyatis akajei* | 1 | 1 | 1 | 3 | 1 | 1 | 0 | 1 | 0 |
| *Dasyatis zugei* | 1 | 1 | 1 | 3 | 1 | 1 | 0 | 1 | 0 |
| *Taeniurops meyeni* | 1 | 0 | 1 | 3 | 1 | 0 | 0 | 1 | 0 |
| *Dasyatis microps* | 1 | 3 | 1 | 3 | 1 | 1 | 0 | 0 | 0 |
| *Neotrygon kuhlii* | 1 | 1 | 1 | 3 | 1 | 0 | 1 | 1 | 0 |
| *Neotrygon leylandii* | 1 | 1 | 1 | 3 | 1 | 0 | 1 | 1 | 0 |
| *Neotrygon annotata* | 1 | 1 | 1 | 3 | 1 | 0 | 1 | 1 | 0 |
| *Taeniura lymma* | 1 | 1 | 1 | 3 | 1 | 0 | 1 | 1 | 0 |
| *Plesiobatis daviesi* | 1 | 2 | 1 | 3 | 1 | 0 | 0 | 0 | 1 |
| *Hexatrygon bickelli* | 1 | 0 | 1 | 3 | 0 | 0 | 0 | 0 | 1 |
| *Urolophus paucimaculatus* | 1 | 0 | 1 | 3 | 1 | 0 | 0 | 0 | 1 |
| *Urolophus cruciatus* | 1 | 0 | 1 | 3 | 1 | 0 | 1 | 0 | 1 |
| *Trygonoptera testacea* | 1 | 0 | 1 | 3 | 1 | 0 | 0 | 0 | 1 |
| *Trygonoptera imitata* | 1 | 0 | 1 | 3 | 1 | 0 | 0 | 0 | 1 |
| *Urobatis jamaicensis* | 1 | 0 | 1 | 3 | 1 | 0 | 1 | 0 | 1 |
| *Urobatis halleri* | 1 | 0 | 1 | 3 | 1 | 0 | 1 | 0 | 1 |
| *Urobatis venezuelae* | 1 | 3 | 1 | 3 | 1 | 0 | 1 | 0 | 1 |
| *Urobatis maculatus* | 1 | 0 | 1 | 3 | 1 | 0 | 1 | 0 | 1 |
| *Urotrygon concentricus* | 1 | 0 | 1 | 3 | 1 | 0 | 1 | 0 | 1 |
| *Potamotrygon motoro* | 1 | 3 | 1 | 3 | 1 | 0 | 1 | 3 | 0 |
| *Potamotrygon falkneri* | 1 | 3 | 1 | 3 | 1 | 0 | 1 | 3 | 0 |
| *Potamotrygon tatianae* | 1 | 3 | 1 | 3 | 1 | 0 | 1 | 3 | 0 |
