# Supplementary material for: Molecular and Morphological Analyses Reveal Phylogenetic Relationships of Stingrays Focusing on the Family Dasyatidae (Myliobatiformes)
Source: PLoS One. 2015 Apr 13;10(4):e0120518. doi: 10.1371/journal.pone.0120518 (PMC4395009; doi:10.1371/journal.pone.0120518)
Supplement: S4 Table — Measurements are expressed as percentage of disc width. (DOCX) [file pone.0120518.s004.docx]

**Table S4.** Morphometric measurements of Himanturidae, Dasyatidae, Neotrygonidae and Pastinachidae. Measurements are expressed as percentage of disc width.

|  | Himanturidae (11 species, 105 samples) | | | | | Dasyatidae (3species, 123 samples) | | | | | Neotrygonidae (3species, 10 samples) | | | | | Pastinachidae (2 species 19 samples) | | | | |
| --- | --- | --- | --- | --- | --- | --- | --- | --- | --- | --- | --- | --- | --- | --- | --- | --- | --- | --- | --- | --- |
| Measurement | **Max** | **Min** | **n** | **Mean** | **S.D.** | **Max** | **Min** | **n** | **Mean** | **S.D.** | **Max** | **Min** | **n** | **Mean** | **S.D.** | **Max** | **Min** | **n** | **Mean** | **S.D.** |
| Disc width, mm | 1920.0 | 61.5 | 73 | 201.3 | 273.1 | 545.0 | 81.0 | 112 | 173.8 | 84.3 | 275.0 | 183.0 | 5 | 221.9 | 40.6 | 747.0 | 235.0 | 4 | 427.0 | 226.9 |
| Total length | 468.0 | 154.7 | 73 | 270.2 | 67.6 | 346.1 | 75.2 | 112 | 277.2 | 49.5 | 238.8 | 163.6 | 5 | 197.3 | 28.3 | 402.9 | 297.4 | 4 | 338.5 | 45.5 |
| Disc length | 120.0 | 82.3 | 73 | 104.1 | 6.0 | 112.9 | 79.9 | 112 | 101.0 | 6.0 | 87.3 | 79.2 | 5 | 84.4 | 3.3 | 97.6 | 82.6 | 4 | 90.4 | 6.4 |
| Pelvic fin length | 26.0 | 11.9 | 63 | 16.1 | 2.4 | 23.1 | 11.1 | 110 | 17.8 | 3.1 | 22.4 | 14.2 | 5 | 19.8 | 3.3 | 26.9 | 20.5 | 4 | 23.2 | 2.8 |
| Pelvic fin base | 19.7 | 9.6 | 65 | 13.1 | 2.1 | 19.9 | 8.6 | 108 | 14.2 | 2.7 | 13.0 | 13.0 | 1 | 13.0 |  | 22.4 | 14.8 | 4 | 18.3 | 3.2 |
| Snout to origin of cloaca | 107.0 | 67.7 | 73 | 88.5 | 6.4 | 103.3 | 66.4 | 112 | 87.9 | 7.3 | 73.4 | 68.2 | 5 | 70.5 | 2.6 | 78.7 | 68.9 | 4 | 74.6 | 4.4 |
| Cloaca origin to tail tip | 376.7 | 59.9 | 63 | 169.5 | 56.7 | 261.7 | 82.6 | 108 | 192.8 | 50.7 | 171.8 | 26.6 | 5 | 94.3 | 63.7 | 324.8 | 175.9 | 4 | 250.6 | 61.3 |
| Tail width, axil to pelvic fins | 12.3 | 4.1 | 73 | 9.2 | 1.6 | 12.3 | 3.7 | 112 | 8.5 | 1.4 | 8.2 | 6.6 | 5 | 7.7 | 0.7 | 11.9 | 9.1 | 4 | 10.9 | 1.3 |
| Tail height, axil to pelvic fins | 9.0 | 3.4 | 73 | 5.9 | 1.1 | 6.8 | 2.8 | 112 | 5.0 | 0.8 | 6.2 | 4.4 | 5 | 5.2 | 0.7 | 7.2 | 5.7 | 4 | 6.5 | 0.6 |
| Head length | 63.0 | 22.3 | 63 | 32.9 | 7.7 | 59.5 | 21.9 | 112 | 30.8 | 5.9 | 42.3 | 16.6 | 5 | 34.8 | 10.3 | 45.8 | 38.2 | 4 | 42.2 | 3.3 |
| Preorbital length | 38.6 | 18.0 | 63 | 31.3 | 3.7 | 40.2 | 17.0 | 110 | 31.5 | 4.9 | 19.8 | 16.4 | 5 | 17.9 | 1.4 | 22.4 | 18.1 | 4 | 20.5 | 1.8 |
| Eye diameter | 6.7 | 1.0 | 73 | 4.3 | 1.1 | 6.6 | 1.8 | 112 | 3.8 | 0.7 | 6.2 | 5.1 | 5 | 5.5 | 0.5 | 3.1 | 1.8 | 4 | 2.6 | 0.6 |
| Interorbital length | 24.6 | 9.3 | 73 | 16.4 | 2.9 | 21.6 | 8.1 | 112 | 17.0 | 2.7 | 16.4 | 8.1 | 5 | 10.3 | 3.4 |  |  |  |  |  |
| Spiracle length | 12.1 | 4.7 | 73 | 6.9 | 1.1 | 10.4 | 5.1 | 112 | 7.5 | 1.1 | 6.7 | 5.2 | 5 | 5.9 | 0.6 | 7.7 | 5.9 | 4 | 6.9 | 0.8 |
| Interspiracular length | 25.8 | 12.9 | 73 | 18.5 | 2.1 | 22.1 | 12.5 | 112 | 18.1 | 2.6 | 15.4 | 12.7 | 5 | 13.8 | 1.1 | 18.7 | 14.3 | 4 | 16.8 | 1.9 |
| Prenarial length | 31.7 | 14.3 | 73 | 25.2 | 3.6 | 33.2 | 13.1 | 110 | 24.7 | 5.1 | 14.4 | 12.2 | 5 | 13.0 | 1.0 | 15.3 | 11.7 | 4 | 13.8 | 1.6 |
| Internarial length | 13.2 | 7.2 | 63 | 11.3 | 1.3 | 13.1 | 8.8 | 110 | 10.6 | 0.7 | 8.5 | 7.0 | 5 | 7.6 | 0.7 |  |  |  |  |  |
| Nasal curtain length | 12.4 | 3.6 | 73 | 5.8 | 1.2 | 10.8 | 4.5 | 112 | 6.0 | 0.6 | 7.2 | 4.4 | 5 | 5.7 | 1.1 | 6.3 | 4.7 | 4 | 5.7 | 0.7 |
| Nostril length | 5.7 | 2.9 | 63 | 4.4 | 0.7 | 5.6 | 3.2 | 110 | 4.3 | 0.4 | 3.9 | 2.6 | 5 | 3.3 | 0.5 | 3.7 | 2.5 | 4 | 3.2 | 0.5 |
| Preoral length | 38.7 | 18.7 | 67 | 30.7 | 3.7 | 39.7 | 16.7 | 112 | 30.4 | 5.6 | 18.8 | 15.9 | 5 | 17.3 | 1.2 | 19.9 | 15.1 | 4 | 17.7 | 2.2 |
| Mouth width | 11.7 | 6.1 | 73 | 9.6 | 1.1 | 10.8 | 7.6 | 112 | 9.2 | 0.6 | 8.1 | 6.4 | 5 | 7.3 | 0.7 | 8.6 | 7.4 | 4 | 8.2 | 0.6 |
| Width of 1st gill slit | 3.9 | 1.4 | 73 | 2.8 | 0.5 | 6.7 | 1.8 | 112 | 3.0 | 0.5 | 3.7 | 2.8 | 5 | 3.2 | 0.4 | 3.3 | 2.8 | 4 | 3.1 | 0.2 |
| Width of 3rd gill slit | 4.0 | 2.1 | 61 | 3.0 | 0.5 | 4.1 | 2.4 | 44 | 3.3 | 0.5 | 3.6 | 2.8 | 5 | 3.2 | 0.3 |  |  |  |  |  |
| Width of 5th gill slit | 2.9 | 1.1 | 71 | 1.9 | 0.4 | 6.1 | 1.1 | 112 | 2.0 | 0.5 | 2.7 | 1.9 | 5 | 2.2 | 0.3 | 2.6 | 2.2 | 4 | 2.4 | 0.2 |
| Distance between 1st gill slits | 30.8 | 14.6 | 73 | 23.8 | 2.4 | 25.0 | 11.7 | 112 | 21.4 | 2.2 | 18.1 | 15.2 | 5 | 16.4 | 1.2 | 21.9 | 17.8 | 4 | 19.8 | 1.8 |
| Distance between 5th gill slits | 19.2 | 9.0 | 73 | 15.8 | 1.8 | 16.0 | 8.3 | 112 | 13.7 | 1.2 | 10.2 | 8.5 | 5 | 9.1 | 0.7 | 13.8 | 12.2 | 4 | 13.1 | 0.7 |
| Ventral tail fold length | 0.0 | 0.0 | 73 | 0.0 | 0.0 | 99.3 | 31.7 | 112 | 56.1 | 18.1 | 77.6 | 54.8 | 5 | 65.4 | 9.5 | 123.4 | 74.0 | 4 | 102.7 | 20.9 |
